# H_2_ production by the photocatalytic reforming of cellulose and raw biomass using Ni, Pd, Pt and Au on titania

**DOI:** 10.1098/rspa.2016.0054

**Published:** 2016-07

**Authors:** A. Caravaca, W. Jones, C. Hardacre, M. Bowker

**Affiliations:** 1 School of Chemistry and Chemical Engineering, Queen’s University Belfast, Belfast BT9 5AG, UK; 2 UK Catalysis Hub, Research Complex at Harwell, Rutherford Appleton Laboratory, Harwell, Oxford OX11 0FA, UK; 3 Cardiff Catalysis Institute, School of Chemistry, Cardiff University, Cardiff CF10 3AT, UK

**Keywords:** photoreforming, cellulose, biomass, fescue grass, hydrogen production

## Abstract

Here, we report a method for sustainable hydrogen production using sunlight and biomass. It is shown that cellulose can be photoreformed to produce hydrogen, even in solid form, by use of metal-loaded titania photocatalysts. The experiments performed verified that the process is enabled by initial hydrolysis via glucose, which itself is shown to be efficiently converted to produce hydrogen by photocatalysis. Importantly, it is shown that not only precious metals such as Pt, Pd and Au can be used as the metal component, but also much more economic and less environmentally damaging Ni is effective. Even more importantly, we show for the first time, to the best our knowledge, that fescue grass as raw biomass can be effective for hydrogen production without significant pre-treatment. This provides additional benefits for the efficiency of biomass hydrogen production, because fewer processing steps for the raw material are required than in the production of purer forms of cellulose, for example.

## 1. Introduction

The number of publications searching for clean energy sources has dramatically increased in recent years owing to the importance of a move from fossil fuel resources to renewable feedstocks [1]. As part of this transition, hydrogen is considered as an important future energy carrier mainly owing to its high energy content and the absence of toxic or greenhouse gases during its combustion [2]. However, hydrogen is present on Earth in combination with other elements and efficient means to effect the transformation of these substances is necessary [3]. In addition, ‘sustainable’ hydrogen is required, which is only generated when the raw material is biomass, which consumes CO_2_ during its growth [4], or via water electrolysis or photolysis.

One method to produce hydrogen from biomass is by photocatalytic reforming (or photoreforming). The aim of this approach is to use solar energy for the activation of the catalysts involved in the reforming of these substances. In fact, the photoreforming process results in an improvement in the photocatalytic water-splitting process by the presence of the organic component as a hole scavenger.

The ideal scenario in this field is the photocatalytic production of H_2_ and O_2_ from liquid-phase water, and it was first studied by Fujishima & Honda in the early 1970s [5]. The main drawbacks of the water-splitting process are the inherently unfavourable thermodynamics, and, consequently, the rapid recombination of the O_2_ and H_2_ produced, limiting hydrogen production [6]. In the absence of suitable charge scavengers, the recombination of electrons (e^−^) and holes (h^+^) generated by the photoirradiation of the semiconductor catalyst takes place. Both the recombination of the reaction products and the recombination of the photo-generated charge carriers dramatically decrease hydrogen production and, therefore, the efficiency of the water-splitting process. In order to overcome these issues, one alternative is to add sacrificial agents to consume the oxygen-derived species produced, avoiding further dioxygen gas evolution and its recombination with H_2_. Moreover, these sacrificial agents act as hole scavengers, diminishing the recombination of e^−^ and h^+^.

The photoreforming of alcohols, such as methanol [6–10], ethanol [11,12] or glycerol [12–15], has been thoroughly studied in the last decade. These alcohols belong to the first generation of biofuels, provided that they are obtained from biomass resources, such as sugars, starches and vegetable oils. However, the utilization of these materials as a hydrogen source competes with their use as food. Hence, the second generation of biofuels has mainly concentrated on the utilization of lignocellulosic biomass, because they could be grown in combination with food or on barren land [16].

Lignocellulose consists of three main polymers, namely lignin (15–20%), hemicellulose (25–35%) and cellulose (40–50%) [17]. Cellulose is the most abundant biopolymer on Earth, and thus its conversion into chemicals and biofuels has attracted great interest [16]. The aim of this work was to study the conversion of cellulose into hydrogen by means of the photoreforming reaction, using cellulose as the sacrificial agent.

The conversion of cellulose usually takes place through its hydrolysis into glucose. Thereafter, the conversion of glucose can give rise to a wide variety of chemicals through mineralization [18], pyrolysis [19], etc. However, even though the production of H_2_ by the photocatalytic reforming of cellulose (or glucose) could enhance the hydrogen economy, this has not been extensively studied previously. Table 1 summarizes the state of the art with respect to the photoreforming of cellulose and glucose solutions for hydrogen production. The initial studies by Kawai & Sakata [20,21] demonstrated the possibility of producing hydrogen directly by irradiation of a TiO_2_-based catalyst with a cellulose/water slurry. Kondarides *et al.* [24] compared the photoreforming of several biomass-derived compounds including cellulose, and highlighted the potential of the photoreforming of cellulose for practical application. Speltini *et al.* [41] undertook a systematic study on the photoreforming of cellulose over a Pt/TiO_2_ catalyst. However, none of these studies have reported a detailed reaction mechanism of the studied process.

**Table 1.** State of the art in cellulose and glucose photoreforming.

sacrificial agent	catalyst	catalyst preparation method	reference
cellulose	8.7% RuO_2_/4.3% Pt/TiO_2_	physical mixture of precursors	Kawai & Sakata [20]
cellulose	5% Pt/TiO_2_	photoelectrochemical deposition	Kawai & Sakata [21]
glucose	12% Pt/0–17% RuO_2_/TiO_2_	Pt colloid + ion-exchangefiltration + deposition overTiO_2_	St John *et al.*[22]
glucose^*a*^	1% M/TiO_2_ (M = Pt, Ru, Au, Pd, Rh, Ag)	wet impregnation	Fu *et al.*[23]
glucose, cellulose^a^	0.5% Pt/TiO_2_	wet impregnation	Kondarides *et al.*[24]
glucose	0.3% M/TiO_2_ (M = Ni, Cu)	wet impregnation	GuoPeng *et al.*[25]
	0.3% M/TiO_2_ (M = Pt, Rh, Ru, Au)	photodeposition	
glucose^a^	Pt/TiO_2_	photodeposition	Luo *et al.*[26]
glucose^a^	0.5%/SrTiO_3_	sol–gel	Puangpetch *et al.*[27]
glucose	Fe_2_O_3_/TiO_2_	—	Hu *et al.*[28]
glucose^b^	TiO_2_	theoretical study	Balducci [29]
glucose^a^	Pt/TiO_2_	photodeposition	Nakatani & Kometani [30]
	Ni/TiO_2_	impregnation	
glucose	ZnS/ZnIn_2_S_4_	solvothermal method	Li *et al.*[31]
glucose^a^	0.1% Pt/TiO_2_	photodeposition	Xu *et al.*[32]
glucose	M/TiO_2_ (M = Pt, Pd)	sol–gel	Colmenares *et al.*[33]
glucose	0.5% Pt/Cd_0.5_Zn_0.5_S	photodeposition	Li *et al.*[34]
glucose^a^	0.2–2% Au/TiO_2_	photodeposition	Gärtner *et al.*[35]
glucose	1% Pt/TiO_2_	photodeposition	Zhou *et al.*[36]
glucose	1% M/TiO_2_ (M = Au, Ag, Cu, Ni, Pd, Rh, Zn)	photodeposition	Gomathisankar *et al.*[37]
glucose	0.2% M/TiO_2_ (M = Rh, Pd, Pt)	photodeposition	Chong *et al.*[38]
	1% M/TiO_2_ (M = Cu, Ni)		
glucose	Cu_2_O	one-step synthesis method	Zhang *et al.*[39]
glucose^a^	X-Fe_2_O_3_ (X = polymorph *α*,*β* and *ε*)	chemical vapour deposition(CVD)	Carraro *et al.*[40]
cellulose	0.5 Pt/TiO_2_	photodeposition	Speltini *et al.*[41]

^a^These publications study the photoreforming of glucose or cellulose together with other sacrificial agents.

^b^Theoretical study about the chemisorption of glucose on TiO_2_.

In contrast (table 1), a much larger number of articles have been published regarding H_2_ production from glucose/water solutions [22–39]. Indeed, most of these have coincided with the significant increase in reports regarding hydrogen production technology and the use of biofuels as alternatives to the use of fossil fuels. Despite the large number of investigations and catalysts used including different metals (Pt, Au, Pd, Ag, Ni, Cu) and supports (TiO_2_, Cu_2_O, Cd_0.5_Zn_0.5_S, Fe_2_O_3_), the reaction mechanism for this process is not very clear. In addition, even though the studies about the photoreforming of cellulose assume that the first step in this process is the hydrolysis towards glucose, none of the publications shown in table 1 compare hydrogen evolution with both molecules over similar catalysts.

In order to enhance our knowledge regarding previous studies [20,21,24,41] about the photocatalytic reforming of cellulose over Pt/TiO_2_-based catalysts, we have studied the parameters that influence the rate of H_2_ production and the rate law for cellulose photoreforming. Moreover, these results are compared for the first time with the rates and the rate law in photoreforming of glucose. In addition, for the first time, we have studied the photoreforming of cellulose over M/TiO_2_ catalysts (M = Pd, Au, Ni) and compared their performance with that in photoreforming of glucose over the same metals. The comparison of the activity using cellulose and glucose as sacrificial agents allows the first step in the photoreforming of cellulose to be elucidated. In this case, it is thought that initially (photo)hydrolysis of cellulose occurs. Moreover, the use of non-precious metals (such as Ni) is of particular interest in view of the further development and practical application of the photoreforming of cellulose, because it is a much more Earth-abundant metal than the precious metals. Finally, in view of the further practical development of this technology, we studied for the first time, to the best of our knowledge, hydrogen production by the photocatalytic reforming of fescue grass as a clean and abundant raw biomass.

## 2. Experimental

The reaction set-up consisted of a 315 cm^3^ Pyrex round-bottomed flask, with a purge line and a septum for sampling. Pyrex glass cuts off all wavelengths of less than 300 nm, preventing high-energy UVB or UVC irradiation from reaching the reaction mixture. All the catalysts were prepared by the wet impregnation method. The required amounts of the metal precursors (H_2_PtCl_6_⋅6 H_2_O, PdCl_2_, NiNO_3_⋅6 H_2_O, HAuCl_4_; all supplied by Sigma-Aldrich^®^) were dissolved in 10 cm^3^ of distilled water (and a few drops of HCl for PdCl_2_). Each solution was mixed with a Degussa (Evonik) P25 TiO_2_ support and stirred under heating at 60°C for 4 h. The resulting slurry was then dried at 150°C for 2 h in air and calcined at 500°C for 2 h. Prior to testing, the catalysts were ground and sieved to ensure that the aggregate size was less than 50 μm. Different metal loadings were prepared for Pt/TiO_2_ catalysts, ranging from 0.2% to 1.6%, whereas Pd/TiO_2_, Ni/TiO_2_ and Au/TiO_2_ catalysts had a loading of approximately 0.2%. The loading of the catalysts was verified by inductively coupled plasma, so that the Pt/TiO_2_ catalysts had a real loading of 0.26%, 0.7%, 1% and 1.6%, and the Pd/TiO_2_, Ni/TiO_2_ and Au/TiO_2_ catalysts had a loading of 0.22%, 0.24% and 0.13%, respectively.

For a typical reaction experiment, 150 mg of catalyst and 200 cm^3^ of deionized water were placed in the reaction set-up, together with a certain amount of the sacrificial agent (microcrystalline cellulose (Alfa Aesar^®^) or glucose (Sigma-Aldrich^®^. The mixture was sonicated for 30 min and then purged with Ar for another 30 min to remove the dissolved oxygen. Finally, under photoirradiation conditions, the reactor was irradiated by a 150 W Xe arc lamp. Gas samples (volume 1 cm^3^) were taken every 30 min, and analysed in a gas chromatograph (Shimadzu 2014) using HayeSep-N and Molsieve (Shimadzu 80–100 mesh) columns in series and a thermal conductivity detector.

Some experiments were carried out with fescue grass as a raw biomass sacrificial agent. It was obtained from a domestic garden, where multipurpose grass seed (Westland Horticulture Ltd) was sown. Prior to the photocatalytic experiment, the grass was thoroughly washed with water and then pure methanol (to extract the chlorophyll) at 55°C in a sonic bath. The grass was then dried at 120°C overnight and ground in a mortar.

Surface area measurements of the above-mentioned catalysts were conducted, using a Micromeritics ASAP system with N_2_ as the sorbate. The total specific surface areas were determined by the multi-point Brunauer–Emmett–Teller (BET) method.

The morphology of the catalysts and the cellulose was characterized by X-ray diffraction (XRD) and transmission electron microscopy (TEM). The XRD spectra were obtained using a PANalytical X’Pert Pro X-ray diffractometer using Cu–K_α_ irradiation. Diffractograms were performed from 10° to 90° with a step size of 0.008°. Crystallite sizes in the different phases were estimated from line broadening of the corresponding XRD peaks by using the Scherrer equation. Anatase–rutile fractions were calculated by taking into account the relative diffraction peak intensities of crystalline planes (101) at approximately 25.3° and (110) at approximately 27.5° of anatase and rutile, respectively [42]. TEM images were recorded in a JEOL-2100 with a LaB_6_ filament microscope operating at a voltage of 200 kV. Catalyst samples were prepared for TEM characterization by dispersing the catalyst powder in high-purity ethanol, followed by sonication for 10 min. A drop of this dispersion was evaporated on a holey carbon film supported by a 300-mesh copper TEM grid. In addition, an aqueous cellulose filtrate was characterized by TEM to determine the presence of smaller particles. In this case, a drop of the filtrate was evaporated on the holey carbon film. The samples were then subjected to bright-field diffraction contrast imaging.

The optical properties of the catalysts and cellulose were characterized by UV–Vis diffuse reflectance spectroscopy in the region from 200 to 800 nm with a UV–Vis spectrophotometer (Shimadzu UV-2600). Finally, cellulose morphology was also characterized by scanning electron microscopy (SEM; JEOL JSM-6610LV).

## 3. Results and discussion

### (a) Characterization

The surface areas of pure TiO_2_ and the metal-loaded TiO_2_ catalysts were assessed using the BET analysis. Table 2 shows typical surface area values for P25 [43] at approximately 50 m^2^ g^−1^. Similar values were obtained for the synthesized catalysts, which demonstrated that there was no discernible loss in surface area after loading the support with different metals (at low loadings), with different Pt loadings or after calcination treatment of the samples at 500°C.

**Table 2.** Structural parameters of TiO_2_ (P25) and TiO_2_-based catalysts according to BET and XRD.

	catalyst							
	TiO_2_ (P25)	0.2% Pt/TiO_2_	0.7% Pt/TiO_2_	1% Pt/TiO_2_	1.6% Pt/TiO_2_	0.2% Au/TiO_2_	0.2% Ni/TiO_2_	0.2% Pd/TiO_2_
BET surface area (m^2^ g^−1^)	44.12	53.14	51.07	50.72	55.62	49.46	51.11	50.91
anatase particle size (nm)	20.03	22.34	23.05	21.21	22.59	21.38	21.43	22.40
rutile particle size (nm)	36.37	35.20	38.19	36.72	33.23	36.72	33.96	36.72
% w/w anatase	83.01	81.41	81.33	82.40	77.50	79.65	80.80	80.61

XRD of the Pt/TiO_2_ catalysts with different loadings and the 0.2% M/TiO_2_ catalysts (M = Au, Pd and Ni) was carried out (not shown here). In none of the analysed samples could the presence of diffraction peaks associated with the metal or metal oxides be detected. This could be due to the relatively low metal loading on TiO_2_ (below the detection limits of the apparatus) [44], or to the small size of the metal particles (less than 5 nm). In addition, table 2 shows the crystallite size of the anatase and rutile phases for all the catalysts, together with the relative amount of anatase and rutile. These parameters were calculated from XRD, as described in the Experimental section. The crystallinity and the phases were not affected by the impregnation of the support with different metals or following calcination at 500°C, as expected. This is in good agreement with previous studies, where the anatase–rutile phase transition occurred at temperatures higher than 600°C [32,45].

**Figure 1.**
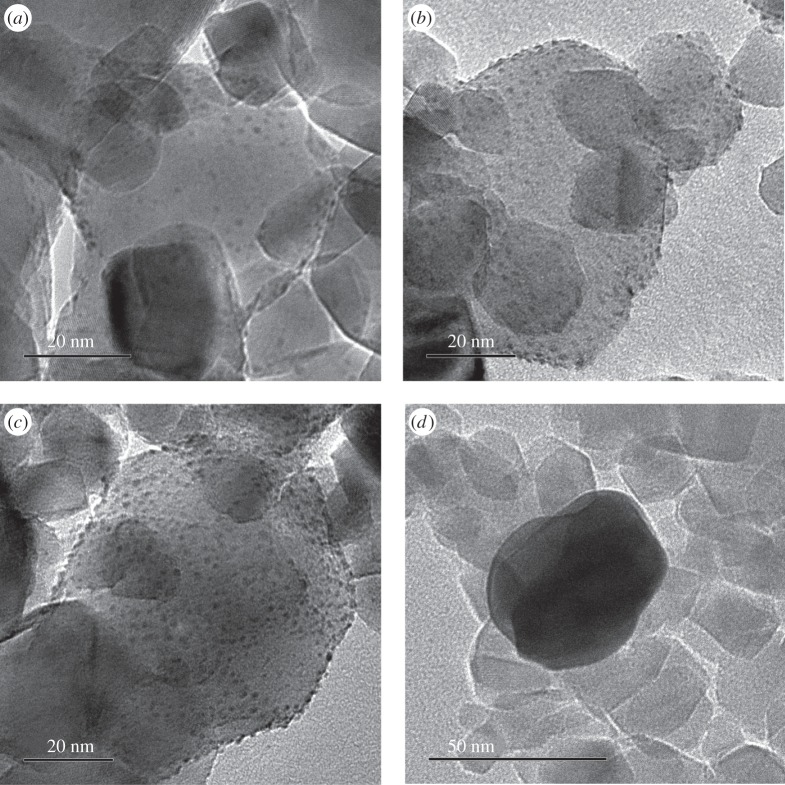
TEM images of 0.7% Pt/TiO_2_ (*a*), 1% Pt/TiO_2_ (*b*), 1.6% Pt/TiO_2_ (*c*) and 0.2% Au/TiO_2_ (*d*).

The morphology of the catalysts was studied by TEM, as shown in figure 1. No metal nanoparticles were observed with 0.2% loadings. However, uniformly distributed metal particles can be distinguished at higher Pt loadings (0.7–1.6% Pt/TiO_2_). The average Pt particle size for these catalysts is approximately 1.5–2.0 nm. In addition, it can be observed that the average Au particle size in the 0.2% Au/TiO_2_ catalyst is much higher than that for the Pt catalysts. These results are in good agreement with previous studies [46,47]. For example, Bamwenda *et al.* [47] observed that the Pt particle size of Pt/TiO_2_ (P25) catalysts was approximately 2 nm and did not change significantly comparing photodeposition, deposition–precipitation and impregnation (similar to the procedure used in this study) preparation methods. However, in the case of Au/TiO_2_ (P25) catalysts, they observed that wet impregnation (in comparison with other preparation methods) yielded much larger particles and poor metal dispersion. We observed a similar behaviour in our Au/TiO_2_ catalyst, with a particle size of approximately 40 nm (although even larger particles were observed). This is probably the reason why wet impregnation is not often used in the preparation of Au/TiO_2_ catalysts.

**Figure 2.**
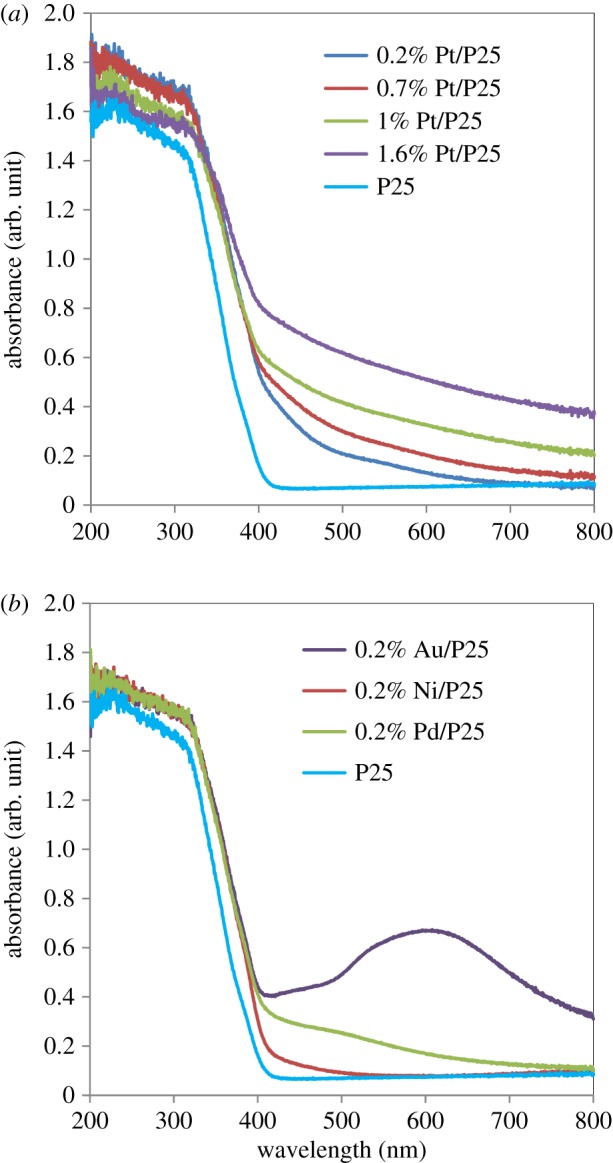
Diffuse reflectance UV–vis spectra for the Pt/TiO_2_ photocatalysts with different metal loadings (*a*), and diffuse reflectance UV–vis spectra for the 0.2% Ni/TiO_2_, 0.2% Au/TiO_2_ and 0.2% Pd/TiO_2_ catalysts (*b*).

The optical properties of all catalysts were characterized by UV–Vis diffuse reflectance spectroscopy. Figure 2*a* shows the UV–Vis absorption spectra for the TiO_2_ support and Pt/TiO_2_ catalysts as a function of metal loading. The absorption edge of TiO_2_ is located at about 400 nm, corresponding to a band gap energy of 3.1 eV. Compared with the pure support, all the Pt/TiO_2_ catalysts exhibit some absorption in the visible region. This is attributed to the surface plasmon resonance of Pt nanoparticles [48]. In addition, an increase in the absorption in the visible range was observed as the Pt loading increased, which can be attributed to the higher amount of metal, as expected.

Figure 2*b* shows the UV–Vis absorption spectra for the Au/TiO_2_, Ni/TiO_2_ and Pd/TiO_2_ catalysts, all with a loading of 0.2%, together with the pure TiO_2_ support. These catalysts show, as for the Pt catalysts, the TiO_2_ absorption band between 200 and 400 nm. Typical absorption in the visible range was observed with the Pd [49] and Au [50] catalysts, with the latter having an absorption band between 500 and 700 nm, which corresponds to the plasmon resonance of Au nanoparticles. This phenomenon is usually observed by a broad absorption peak centred at approximately 520 nm for small nanoparticles (less than 20 nm) [51]. However, the Au/TiO_2_ catalyst prepared in this study exhibits a peak centred at approximately 590 nm. It could be attributed to the larger particle size of the Au particles in our Au/TiO_2_ catalyst, because the absorption band width is known to increase with particle size [51,52]. However, as can be observed in figure 2*a*,*b*, the presence of metal nanoparticles (Pt, Pd, Ni and Au) does not significantly alter the band gap of TiO_2_.

**Figure 3.**
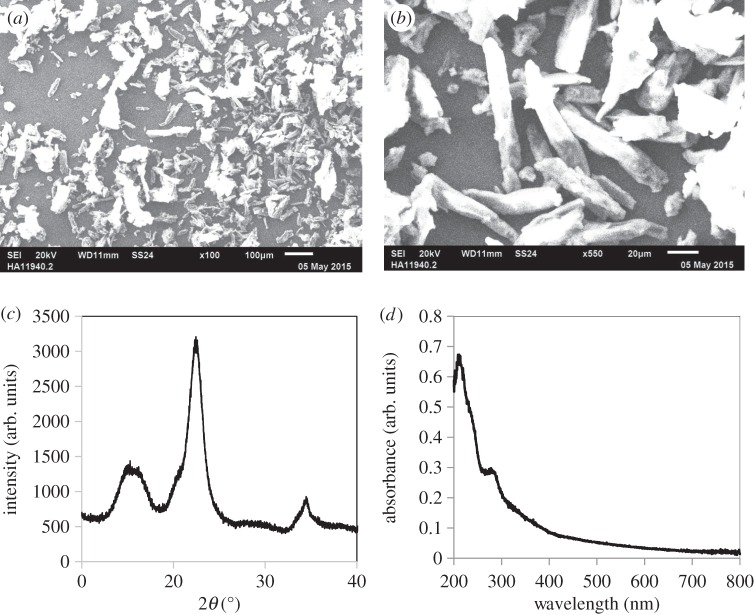
Characterization of microcrystalline cellulose. (*a*,*b*) Scanning electron microscopy images. (*c*) X-ray diffractogram and (*d*) diffuse reflectance UV–vis spectra.

The structure of the microcrystalline cellulose determined by SEM, XRD and diffuse reflectance UV–vis is shown in figure 3. SEM micrographs (figure 3*a*,*b*) show an average particle size of approximately 75 μm together with a fraction of smaller particles. Moreover, the X-ray diffractogram (figure 3*c*) showed a native ‘cellulose I’ structure, with typical peaks centred at 14.8°, 16.3° and 22.6° [53,54]. No other peaks were observed, indicating that the cellulose used was monophasic. The crystallinity index (CrI) of cellulose was 76% according to [54,55]

3.1 $$\text{CrI}(\%)=\frac{I_{002}-I_{\mathrm{AM}}}{I_{002}}\times 100,$$

where *I*_002_ represents the maximum intensity of the (002) lattice diffraction (2*θ*=22.5°) and *I*_AM_ represents the intensity of diffraction scatter for the amorphous part at 2*θ*=18°. Among all the relevant structural parameters to convert cellulose, CrI is a major factor, because a high degree of crystallinity would lead to a difficult interaction of the catalyst with the interior sites of the crystal [55]. In other words, a high degree of crystallinity would lead to slow hydrolysis of cellulose. Hence, the use of microcrystalline cellulose with a CrI of less than 80% seems to be advantageous compared with other common cellulose substrates, such as the native cotton cellulose, with CrI = 80–100%.

The UV–Vis absorption spectra of cellulose (figure 3*d*) showed no absorption in the visible region, although a small amount of absorption in the UV spectra could be observed at wavelengths less than 400 nm. However, compared with the pure TiO_2_ or the TiO_2_-based catalysts, the UV absorption was negligible. This observation, together with the fact that the Pyrex reactor cuts off radiation of less than 300 nm, indicates that any reaction observed is likely to be due to photocatalysis, rather than the photochemical degradation of cellulose.

### (b) Photocatalytic experiments

**Figure 4.**
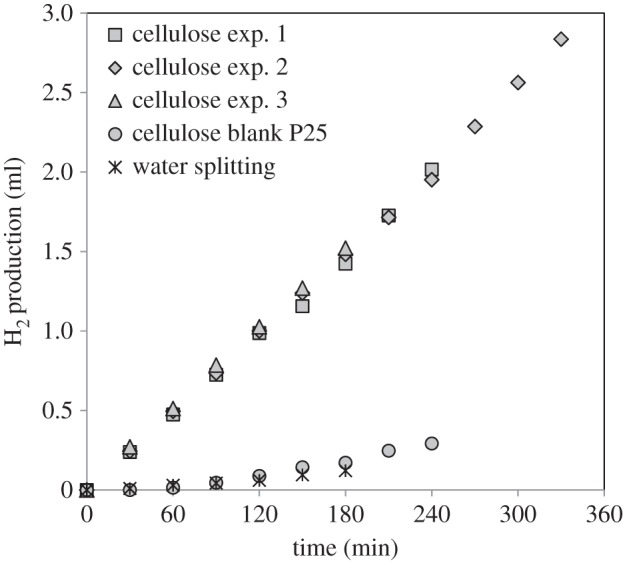
Hydrogen evolution from photocatalytic reforming of cellulose. Reaction conditions—*cellulose exp. 1–3*: 0.2 g cellulose per 200 ml H_2_O, 150 mg 0.2% Pt/TiO_2_, temperature = 60°C. Experiment 1=4 h, experiment 2=5.5 h, experiment 3=3 h; *cellulose blank P25*: 0.2 g cellulose per 200 ml H_2_O, 150 mg TiO_2_ (P25), temperature = 60°C; *water-splitting*: 200 ml H_2_O, 150 mg 0.2% Pt/TiO_2_, temperature = 60°C.

Figure 4 shows the H_2_ evolution (in cm^3^) as a function of irradiation time from a cellulose/water dispersion using a 0.2% Pt/TiO_2_ catalyst. Three separate experiments are shown to demonstrate the reproducibility of the reaction. For comparison, the reaction was also studied over the TiO_2_ without metal loading, as well as over the 0.2% Pt/TiO_2_ in the absence of cellulose to check for any hydrogen production.

It is clear that in the presence of cellulose the hydrogen production is significantly higher than that from water alone and that the photoreforming experiment is highly reproducible. In addition, metal loading seemed to be crucial to achieve high rates. As found previously [20,21,24,41], cellulose behaves as a very efficient sacrificial donor, decreasing the recombination of the photo-generated electrons and holes, by providing an efficient alternative pathway for hole removal. It is somewhat surprising that a solid material such as cellulose can perform so well; this point is addressed below.

The effect of the cellulose is observed only in the presence of the metal nanoparticles, with the pure TiO_2_ showing negligible rates similar to those observed in the absence of a sacrificial agent. Other studies of photoreforming processes, including those from our own group, indicate that the metal can play a number of different roles. The nanoparticles can prolong the lifetime of the photo-generated charge carriers (e^−^ and h^+^) by acting as electron sinks by creating a Schottky barrier between the metal and the TiO_2_ semiconductor [9]. In addition, there is also a catalytic role of the Pt nanoparticles. For example, in the photoreforming of methanol [6,56], the addition of noble metals such as Pt, Pd or Ru leads to dehydrogenation reactions in the absence of light-producing CO, but which then tends to act as a surface poison. On UV irradiation, highly activated oxygen species are produced, which oxidize the CO into CO_2_, thus cleaning the surface. The combination of these effects is responsible for the observed enhancement in hydrogen production on addition of the Pt. At this point, it is worth noting that even a very small loading of Pt (0.2%) led to a large increase in the photoreforming activity of cellulose, which is very interesting from an economical point of view, and even lower metal loadings have been shown to be effective for methanol photoreforming [57].

**Figure 5.**
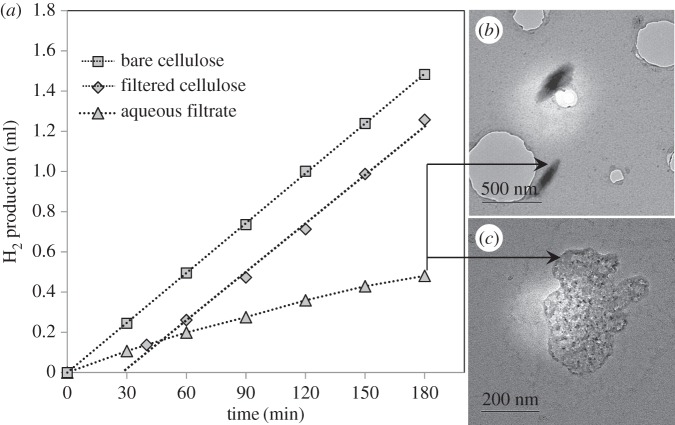
H_2_ evolution after pre-treatment of cellulose and its comparison with that for bare cellulose (*a*). TEM images of the aqueous filtrate (*b*,*c*). Pre-treatment: 3.5 h mixing cellulose/water + filtration by gravity + syringe filtration (0.45 μm). Reaction conditions: *bare cellulose/filtered cellulose*: 0.2 g cellulose per 200 ml H_2_O, 150 mg 0.2% Pt/TiO_2_, temperature = 60°C. *Aqueous filtrate*: 200 ml aqueous filtrate, 150 mg 0.2% Pt/TiO_2_, temperature = 60°C.

In order to investigate whether the hydrogen was produced from the solid cellulose itself or from a water-soluble impurity/by-product present in cellulose, a pre-treatment step was carried out by stirring the cellulose in water for 3.5 h, followed by filtration in two steps (the second step of filtration was limited to particles bigger than 0.45 μm) to remove any water-soluble species. Thereafter, both the aqueous filtrate and the filtered cellulose were tested separately for hydrogen production over the 0.2% Pt/TiO_2_ catalyst. Figure 5*a* shows the hydrogen evolution from these experiments, together with that from the untreated cellulose (figure 4). The hydrogen production by the photoreforming of the washed cellulose was much higher than that from the aqueous filtrate. However, a short induction period of 30 min was observed using the treated cellulose and, thereafter, the hydrogen evolution followed a linear trend. This induction period was not observed with untreated cellulose, although it is worth noting that the rates for the treated and untreated cellulose were very similar after a 30 min reaction. Moreover, the activity when using the aqueous filtrate was much lower but still significant, which could be owing to either a solubilized component or small cellulose particles that pass through the filter. In spite of this, these experiments demonstrate that solid cellulose was the main source of the hydrogen production observed in the previous experiments.

The presence of small particles (less than 0.5 μm) was observed by TEM in the aqueous filtrate (figure 5*b*,*c*). These particles are probably small cellulose particles that passed through the filter. Moreover, in an attempt to weigh the amount of small particles/soluble impurities, 100 ml of the aqueous filtrate was evaporated at approximately 75°C for 3 days in a beaker. After water was evaporated, approximately 2 mg of substances remained. Hence, it may be that these small (cellulose) particles or a small amount of soluble impurities could be responsible for the initial hydrogen production activity. The induction period observed for the filtered cellulose is thought to be due to the reduction of the Pt nanoparticles, although it could also be attributed to the slower initial hydrolysis of the filtered cellulose owing to its higher particle size compared with the untreated cellulose. As observed for the aqueous filtrate-only reaction, hydrogen is produced from the start of the reaction, probably owing to the higher activity of these small particles/soluble impurities. They can reduce the Pt rapidly, leading to high activity from the start of the irradiation in the case of the untreated cellulose, and an induction period after they have been separated.

**Figure 6.**
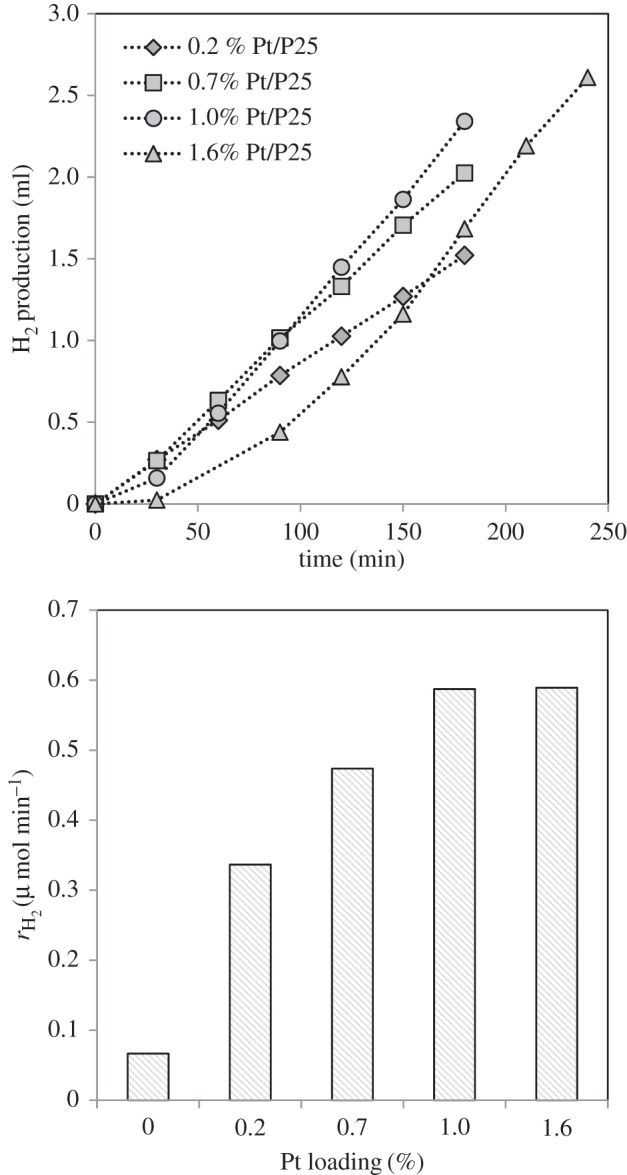
Effect of Pt loading on hydrogen evolution for cellulose photoreforming (*a*) and rate of hydrogen production versus Pt loading (*b*). Reaction conditions: 0.2 g cellulose per 200 ml H_2_O, 150 mg catalyst, temperature = 60°C.

Figure 6 shows the effect of Pt loading on hydrogen evolution (figure 6*a*) and the hydrogen production rate (figure 6*b*) in the cellulose photoreforming process. Catalysts with Pt loadings from 0.2% to 1.6% were studied. Figure 6*a* shows that, for loadings less than or equal to 0.7%, no induction period is observed; however, for higher loadings, an induction period is seen which becomes more pronounced at the highest loading. In all cases, following the induction period, the hydrogen evolution followed a linear trend. This increase in the induction period is attributed to the longer time needed for reduction of Pt at higher loadings [9]. A similar effect has also been previously observed for Pd/TiO_2_ catalysts in the methanol photoreforming reaction [6]. However, the induction period could be avoided if the catalyst is pre-reduced before the photocatalytic experiments, although the possibility to reduce the catalyst *in situ* by the presence of electron-donor molecules (cellulose in this study) seems to be advantageous from a practical point of view. Figure 6*b* shows that, as the loading increases, the hydrogen production rate increases initially before plateauing at loadings greater than or equal to 1%.

A similar dependence of the photocatalytic reaction rate and the loading of Pt has been previously reported for photoreforming of several sacrificial agents, including methanol [48] or glucose [23], where optimum Pt loadings of approximately 1% were found. This dependence may be attributed to the fact that Pt can act as an electron sink, as previously explained. As the Pt nanoparticle loading increases, the electron traps increase, resulting in a higher photocatalytic activity towards hydrogen production owing to a more efficient separation of the photo-generated electrons and holes. However, for Pt loadings of more than 1%, the TiO_2_ surface may be covered by an excess of Pt, resulting in the Pt nanoparticles acting as centres for the recombination of e^−^ and h^+^, with a subsequent detrimental effect on the photocatalytic activity [48]. In addition, as the loading of Pt increases, the penetration of light into the particles decreases, which reduces the number of e^−^/h^+^ pairs.

Other factors to take into account regarding the influence of metal loading are related to the catalytic properties of the metal. For example, an increase in the Pt loading generally leads to a decrease in metal dispersion [23], which might in some cases result in a decrease in its catalytic activity. However, the TEM pictures for Pt/TiO_2_ catalysts with different loadings (figure 1) did not show important variations in the Pt particle size, even though the amount of Pt particles increased with the metal loading, as expected. Therefore, it is unlikely that the dispersion of Pt is the reason that optimum metal loading was found. However, the active sites for some of the reactions involved are thought to be located at the periphery of the metal nanoparticles, i.e. at the metal/TiO_2_ interface. Therefore, as the Pt loading increased, the perimeter length (where the active sites are) is increased up to a certain size. Thereafter, once the particles get too big, the number of interface sites may diminish, because the particles begin to touch [58], which together with the electron sink ability of the Pt nanoparticles could also explain the optimum Pt loading observed.

**Figure 7.**
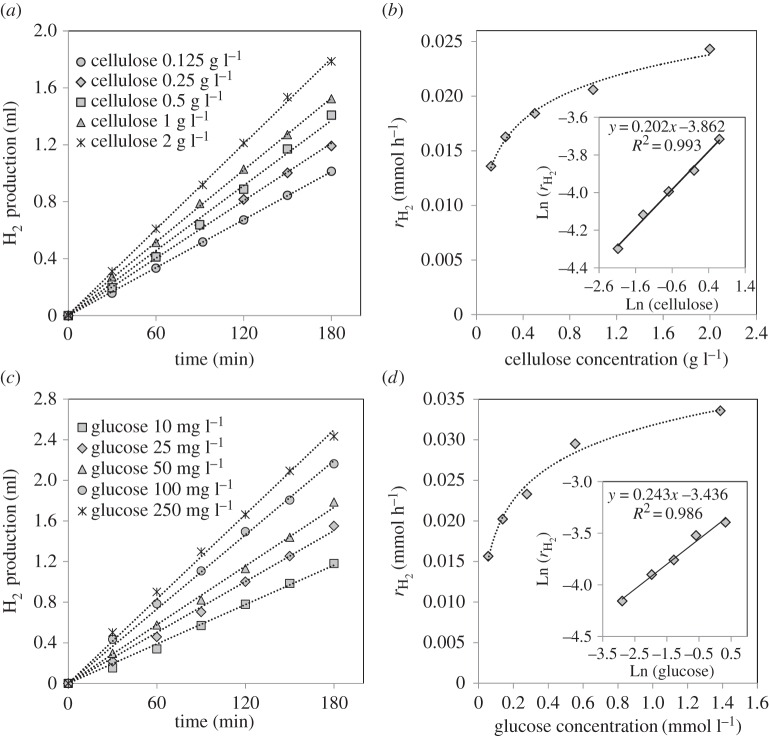
Effect of cellulose and glucose initial concentration on H_2_ production (*a*,*c*), and the rate of H_2_ (*r*_H_2__) versus cellulose and glucose initial concentration (*b*,*d*). The inset figures show the fitting plot of Ln(*r*_H_2__) versus LnC (C = initial concentration of cellulose/glucose). Dotted lines are to guide the eye. Reaction conditions: 200 ml H_2_O, 150 mg 0.2% Pt/TiO_2_, temperature = 60°C.

As the monomer of cellulose, glucose is generally considered to be the key intermediate for cellulose conversion. If this is the case for photoreforming of cellulose, and the hydrolysis step is fast enough, the reaction rate and rate order with respect to cellulose and glucose should be similar. Figure 7*a*,*b* shows the hydrogen evolution and the hydrogen production rates for different cellulose concentrations over the 0.2% Pt/TiO_2_ catalyst. As expected, the hydrogen evolution increases with increasing the initial cellulose concentration [41]. However, a 10-fold increase in cellulose resulted in only a twofold increase in the hydrogen production rate, which suggests a near-zero rate order for cellulose. A very similar trend was observed for the glucose photoreforming reaction over the same catalyst (figure 7*c*,*d*), which has also been observed previously [23,37]. The overall reaction of glucose (3.2) and cellulose (3.3) with water over the catalyst studied can be understood as follows:

3.2 $${\text{C}}_{6}{\text{H}}_{12}{\text{O}}_{6}+6{\text{H}}_{2}\text{O}\to 6{\text{CO}}_{2}+12{\text{H}}_{2}(2)$$

and

3.3 $$({\text{C}}_{6}{\text{H}}_{10}{\text{O}}_{5})n+7n\;{\text{H}}_{2}\text{O}\to 6n\;{\text{CO}}_{2}+12n\;{\text{H}}_{2}.$$

Considering that the above-mentioned reactions took place in a batch system, the overall rate law for these processes can be expressed as

3.4 $$-r_{A}=\frac{r_{H_{2}}}{12n}=kC_{A}^{a}C_{H_{2}O}^{b},$$

where *A* is either cellulose or glucose, *a* and *b* are the reaction orders with respect to cellulose/glucose and water, respectively, *n* varies from 1 (glucose) to the corresponding degree of polymerization of cellulose, and *k* is the rate constant. Equation (3.4) could be simplified considering that, owing to the excess of water, the water concentration remains unchanged,

3.5 $$r_{H_{2}}=k_{\mathrm{obs}}C_{A}^{a},$$

where $k_{\mathrm{obs}}=12nKC_{H_{2}O}^{b}$. According to equation (3.5), the representation of Ln(*r*_H_2__) versus Ln(*C*_*A*_) leads to a linear fit, and its slope corresponds to the reaction order ‘a’ with respect to cellulose (inset in figure 7*b*) and glucose (inset in figure 7*d*). These figures display very close reaction orders for both sacrificial agents (*a*_glucose_=0.24, *a*_cellulose_=0.2). In previous studies of the photoreforming of cellulose [20,21,24,41], it was assumed that the cellulose photoreforming process involves *in situ* hydrolysis under the influence of irradiation, giving rise to the production of glucose. Thereafter, glucose is likely to be reformed under irradiation to produce hydrogen. This theory is in good agreement with the results shown in figure 7. The fact that the reaction orders with respect to cellulose and glucose are very similar indicates that glucose might be, for both processes, the main agent responsible for the hydrogen produced. If this is the case, the hydrolysis step should be faster than the subsequent photoreforming of glucose, it not being, therefore, the rate-determining step of the overall process, as shown in the following equation:

3.6 $$({\text{C}}_{6}{\text{H}}_{10}{\text{O}}_{5})n+7n\;{\text{H}}_{2}\text{O}\to n{\text{C}}_{6}{\text{H}}_{12}{\text{O}}_{6}+6n\;{\text{H}}_{2}\text{O}\to 6n\;{\text{CO}}_{2}+12n\;{\text{H}}_{2}.$$

**Figure 8.**
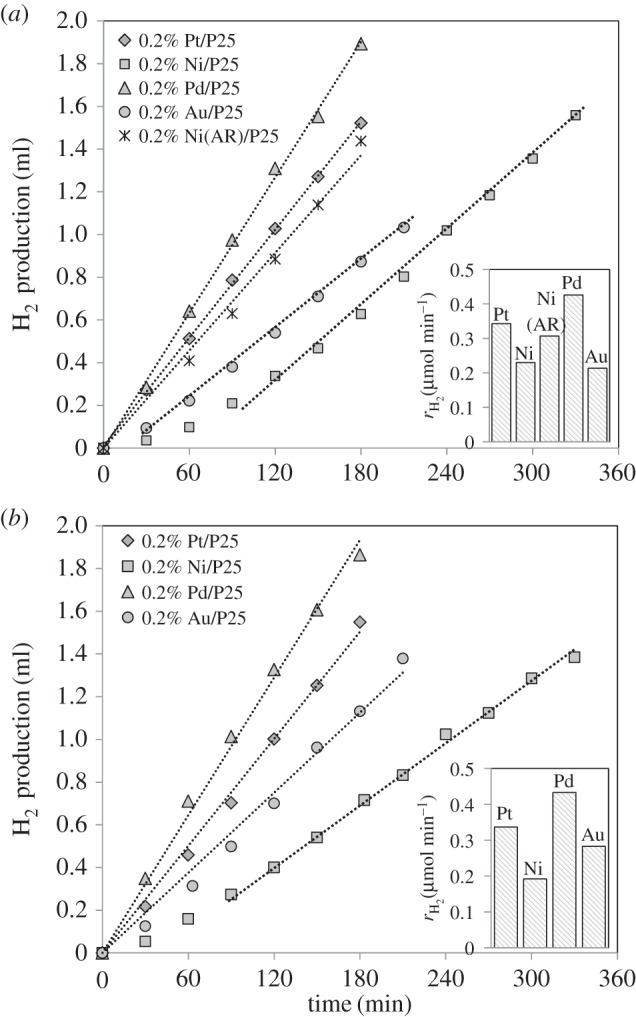
Influence of the co-catalyst on the hydrogen evolution for cellulose photoreforming (*a*) and for glucose photoreforming (*b*). Reaction conditions: 200 mg cellulose (*a*) and 25 mg l^−1^ glucose (*b*), 200 ml H_2_O, 150 mg catalyst, temperature = 60°C. The inset figure shows the H_2_ production rate for each catalyst.

The influence of the choice of metal which is supported on TiO_2_ was studied for the photocatalytic reforming of cellulose. Figure 8*a* shows the hydrogen evolution together with the hydrogen production rates over Pd/TiO_2_, Au/TiO_2_, Ni/TiO_2_ and Pt/TiO_2_ (previously shown in figure 4), all with a metal loading of approximately 0.2%. Figure 8*b* shows the data for the same catalysts in the photoreforming of glucose for further comparison. A glucose concentration of 25 mg l^−1^ was used due to the fact that, as observed in figure 7, it led to a very similar hydrogen production rate to the photoreforming of 1 g l^−1^ cellulose over the 0.2% Pt/TiO_2_ catalyst.

All catalysts showed significant activity towards the production of hydrogen from cellulose with the hydrogen production rate following the trend Pd>Pt>Ni∼Au after the induction periods. Most notably, a large induction period (90 min) was observed with the Ni/TiO_2_ catalyst, which is to be expected if this is associated with the reduction of the metal as Ni is the most difficult to reduce, as described recently [9]. All previous studies concerning cellulose photoreforming have been carried out over Pt/TiO_2_ and Pt-RuO_2_/TiO_2_ catalysts [20,21,24,41]. This is the first time that this process has been studied over different catalytic materials. It is worth noting that, although the Ni-based catalyst exhibits a long induction time, the photocatalytic activity towards hydrogen production was of the same magnitude as that found for the precious metals (Pt, Pd, Au). In addition, this induction time can be avoided if the metal is pre-reduced prior to the photocatalytic reaction [9]. In order to demonstrate this, the Ni/TiO_2_ catalyst was pre-reduced at 400°C for 1 h (H_2_/He=12.5%, total flow = 40 ml min^−1^) prior to the cellulose photoreforming reaction. The pre-treated catalyst was used under the same reaction conditions (denoted as 0.2% Ni(AR)/P25, where AR stands for ‘after reduction’), and the hydrogen production rate was very similar to that for the as-prepared Ni catalyst following the induction period. These results are important for further practical developments of this process. Moreover, the use of Ni catalysts with very low loadings (0.2%) may lead to significant cost savings compared with the use of noble metal catalysts, enhancing the economy of this process.

In the photoreforming of glucose (figure 8*b*), a similar trend was observed as a function of the catalyst type, as found for cellulose (figure 8*a*). The trend was also found to be in good agreement with previous studies performed over different M/TiO_2_ catalysts (M = Pt, Au, Pd, Ni, Cu, etc.) [25,37] for glucose photoreforming. As previously discussed, hydrolysis of cellulose into glucose is likely to be the first step in the photoreforming process. The photocatalytic results obtained with different metals deposited on the TiO_2_ semiconductor also support this theory. The variation with each metal is more complex owing to the interplay of electronic and catalytic factors. Interestingly, the substantially reduced activity for the gold-based catalyst may suggest that the catalytic effect, for example in the initial dehydrogenation reaction, is important, with electronic factors playing a smaller role.

**Figure 9.**
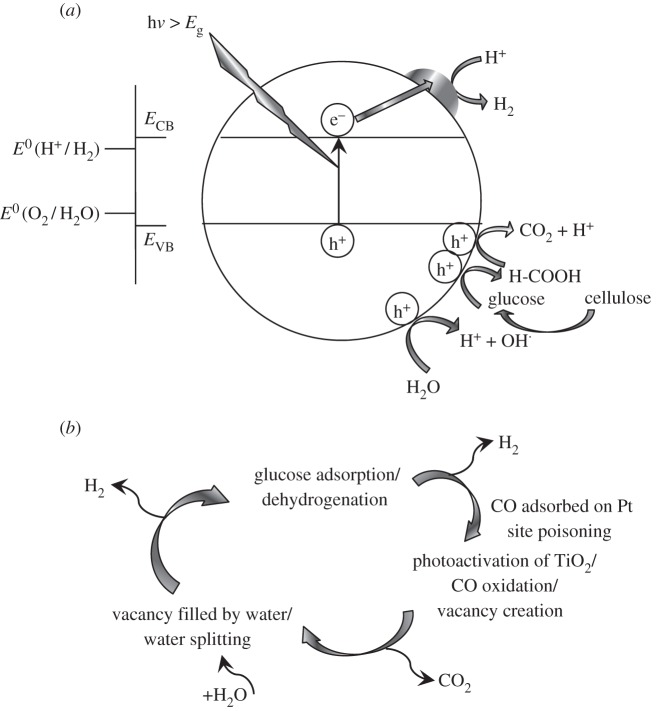
Schematic model of the photocatalytic reforming of cellulose on metal/TiO_2_ catalysts. (*a*) The role of the metal as e^−^ sink to enhance the separation of photo-generated e^−^/h^+^ pairs. (*b*) The catalytic role of the metal in glucose dehydrogenation.

The results obtained in this study support the likelihood of a first hydrolysis step in the photocatalytic reforming of cellulose. Combining the current information in the literature for the photocatalytic reforming of glucose with this study, the following reaction mechanism for photocatalytic reforming of cellulose is proposed (figure 9*a*).

As in every other photocatalytic process over semiconductor materials, the first step is the absorption of a photon with an energy equal to or higher than its band gap (*E*_g_=3.1 eV, figure 2). This leads to the promotion of an electron from the valence band to the conduction band, generating therefore an e^−^/h^+^ pair. As previously explained, Pt or any other metal deposited over the TiO_2_ surface can act as an electron sink, enhancing the separation of the photo-generated charge carriers, and partially avoiding the recombination of electrons and holes. The charge carriers may promote, in the first place, the water-splitting reactions [41]

3.7 $${\text{H}}_{2}\text{O}+{\text{h}}^{+}\to {\text{OH}}^{\cdot }+{\text{H}}^{+}$$

and

3.8 $$2{\text{H}}^{+}+2{\text{e}}^{-}\to {\text{H}}_{2}.$$

Reaction (3.7) might be promoted by the hole carriers, whereas reaction (3.8) would take place at the metal surface. Then, based on figures 7 and 8, cellulose is hydrolysed to produce glucose. In order to investigate whether this step was catalytic or photocatalytic, a reaction was performed in the dark under the same reaction conditions. As expected, no hydrogen was produced under these conditions. Moreover, the liquid product was analysed by high-performance liquid chromatography, and no glucose was observed. This indicates that the hydrolysis of cellulose may take place under photoirradiation. Therefore, the H^+^ and OH species produced by reaction (3.7) can lead to the production of glucose [41], according to

3.9 $${\left({\text{C}}_{6}{\text{H}}_{10}{\text{O}}_{5}\right)}_{n}+{\text{H}}^{+}+{\text{OH}}^{\cdot }\to n{\text{C}}_{6}{\text{H}}_{12}{\text{O}}_{6}.$$

Then, the glucose produced may follow different pathways to produce hydrogen. A specific reaction mechanism for photocatalytic reforming of glucose has not been reported to date. For example, Fu *et al.* [23] proposed that glucose is first adsorbed dissociatively, followed by its subsequent oxidation in a photo-generated hole. The radical produced would attack another glucose, leading to the formation of R-CHO molecules, which are further oxidized to R-COOH with the OH^⋅^ species produced by reaction (3.7). Finally, R-COOH species are photo-oxidized in a hole to produce CO_2_ via a photo-Kolbe reaction. On the other hand, Chong *et al.* [38] described the formation of arabinose, erythrose, glyceraldehyde, gluconic acid and formic acid (together with CO and CO_2_ gas) in the photoreforming of glucose over Rh/TiO_2_ (anatase, rutile and P25) catalysts. They suggested that, by *α*-scission, glucose is transformed through a tandem reaction. First, glucose may be transformed into arabinose, then into erythrose and finally into glyceraldehyde. All these steps take place by oxidation with OH^⋅^ radicals, leading to the production of formic acid and hydrogen. Formic acid might be transformed into CO or CO_2_ as previously explained. Wu *et al.* [25] suggested that glucose- and hydrogen-trapped holes form various oxygen-containing species, leading to the formation of formic acid, and then to the production of CO/CO_2_ and H_2_. Gomathisankar *et al.* [37] proposed, based on other studies of the photo-mineralization or photo-oxidation of glucose, that glucose molecules react with the OH^⋅^ radicals (reaction (3.7)) to generate gluconic acid. The latter would further react with more OH^⋅^ radicals to form C_5_ compounds and formic acid. C_5_ species would then be degraded into C_4_, and C_3_, with the subsequent production of more formic acid and then CO_2_. Speltini *et al.* [41] proposed that hydroxymethylfurfural might be one of the main reaction intermediates based on a previous study of the photo-oxidation of cellulose over TiO_2_ catalysts [59]. Although, to date, the mechanism for the photoreforming of glucose is not completely understood, all the studies describe a continuous degradation/oxidation pathway taking place at the photo-generated holes, leading to the production of formic acid and then a mixture of CO/CO_2_.

A different mechanism could be proposed that takes into account the catalytic role of the metal (figure 9*b*). For instance, previous studies on the photoreforming of methanol or other alcohols [6,8,13,56–58] have suggested that the important dehydrogenation reactions take place on the metal active sites, which would be blocked by the formation and strong chemisorption of CO. Then, by photoirradiation, owing to the TiO_2_ band gap excitation, highly activated oxygen species would be produced, which can then react with CO at the interface between the metal nanoparticles and the oxide support, forming CO_2_.

A similar model could be proposed for the photoreforming of cellulose/glucose. Once the glucose is produced by the (photo)hydrolysis of cellulose, it could be adsorbed and dehydrogenated on the metal nanoparticles. Glucose belongs to a family of oxygenated hydrocarbons (such as ethylene glycol) with a C : O ratio of 1 : 1 [60]. These compounds can produce CO and H_2_, as shown in the following reaction:

3.10 $${\text{C}}_{6}{\text{H}}_{12}{\text{O}}_{6}\to 6\text{CO}+6{\text{H}}_{2}.$$

This reaction takes place on metal catalysts such as Pt via cleavage of C–C bonds, as was proposed in previous studies regarding the aqueous-phase reforming of glucose [61,62]. This reaction would take place in the dark, and in the absence of light, no further reaction would take place owing to the self-poisoning of Pt by adsorbed CO. However, irradiation of TiO_2_ with UV light caused e^−^/h^+^ pairs to be formed, with the hole being highly electrophilic O^−^ [57]. These oxygen species attacked the CO adsorbed in Pt, leading to the production of CO_2_ and a subsequent free active Pt site for the adsorption of more glucose. The anion vacancy on the TiO_2_ support would then be filled by water, liberating more hydrogen. The process is summarized in the following reaction scheme:

3.11 $$\begin{array}{rl} & {\text{C}}_{6}{\text{H}}_{12}{\text{O}}_{6}+{\text{Pt}}_{s}\to 6{\text{CO}}_{\mathrm{ad}}+6{\text{H}}_{2}, \end{array}$$

3.12 $$\begin{array}{rl} & {\text{TiO}}_{2}+\text{h}\nu \to {\text{Ti}}^{3+}+{\text{O}}^{-}+{\text{O}}^{2-}, \end{array}$$

3.13 $$\begin{array}{rl} & {\text{CO}}_{\mathrm{ad}}+{\text{O}}^{-}\to {\text{CO}}_{2}+{\text{Pt}}_{s}+V_{O}^{-} \end{array}$$

3.14 $$\begin{array}{rl} \;\text{and}\; & {\text{V}}_{O}^{-}+{\text{Ti}}^{3+}+{\text{O}}^{2-}+{\text{H}}_{2}\text{O}\to {\text{TiO}}_{2}+{\text{H}}_{2}. \end{array}$$

The former reaction mechanism (figure 9*a*) considered the metal as an electron sink to improve the separation of the photo-generated charge carriers. However, the latter mechanism (figure 9*b*) describes the important catalytic role of Pt itself in the initiation of glucose dehydrogenation. Both processes must take place simultaneously at steady state.

**Figure 10.**
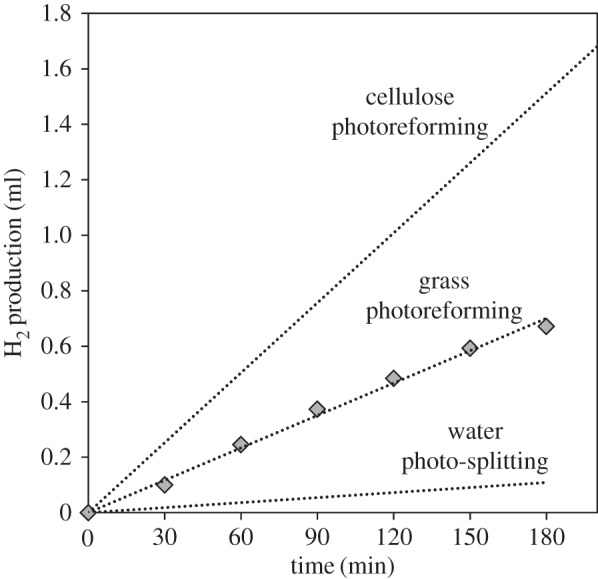
Hydrogen evolution from photocatalytic reforming of grass (and comparison with photocatalytic reforming of cellulose and photocatalytic water-splitting; figure 4). Pre-treatment of grass: washing at 55°C with H_2_O and then with methanol, and drying overnight at 120°C. Reaction conditions: 0.36 g of washed and dried grass per 200 ml H_2_O, 150 mg 0.2% Pt/TiO_2_, temperature = 60°C.

Because the results obtained for cellulose photoreforming are very promising, the possibility of using raw cellulosic biomass was studied. *Fescue grass* was chosen owing to its large availability and high cellulose content (approx. 30% [63]). The grass was pre-treated following the procedure explained in the Experimental section. Then, 360 mg of the washed and dried grass was placed in a photocatalytic reactor, together with the 0.2% Pt/TiO_2_ catalyst used in previous experiments. Figure 10 shows the results obtained for photocatalytic reforming of this raw biomass, together with the results previously shown for photoreforming of cellulose and the reaction in water only with the same catalyst for comparison (figure 4).

As observed in figure 10, significant hydrogen yields were obtained for the photoreforming of grass, about half as much as for pure microcrystalline cellulose, and much higher than in water alone. This demonstrates that this biomass resource behaves as a suitable hole scavenger, strongly enhancing hydrogen production, and showing high potential for the practical development of the photoreforming of biomass resources. This is the first time, to the best our knowledge, that this kind of raw biomass (fescue grass) has been used for hydrogen production in this way. This is a very important finding, because it avoids the processing and separation steps required to make purified cellulose, therefore leading to important cost savings and enhancing the overall hydrogen economy by the photoreforming of biomass.

## 4. Conclusion

In this study, we have clearly demonstrated that hydrogen can be produced by photoreforming of cellulose/water mixtures over TiO_2_ catalysts loaded with metal nanoparticles. Importantly, catalysts based on non-noble metals as well as platinum group metal materials are very effective. For example, the use of much less expensive Ni has been shown to be highly active for hydrogen production, particularly if a pre-reduction step is performed. It is proposed, based on the study of the rate law for cellulose and glucose, that the first step in the photoreforming of cellulose is the (photo)hydrolysis of cellulose into glucose, with the latter then undergoing reforming to hydrogen and CO_2_. To further illustrate the practicality of the process, fescue grass has been shown to be a suitable raw biomass sacrificial donor, producing a significant amount of H_2_ by photoreforming. This process shows high potential for efficient hydrogen economy, because it allows the production of hydrogen directly from the main component of biomass (cellulose), avoiding any intermediate transformation steps (fermentation, etc.) over low loading metal/TiO_2_ catalysts.

## Data accessibility

Information about the data that underpin the results reported in this article, including how to access them, can be found in Cardiff University’s data catalogue at opendata@cardiff.ac.uk.
